# C-951-05. Impact of Early Stress Hyperglycemia in Non-diabetic Burn Patients on Infectious Complications and Mortality

**DOI:** 10.1093/jbcr/irag033.113

**Published:** 2026-04-06

**Authors:** Tuan D Le, Arthur Kom Sipowa, Jacob Furcolo, Melissa M McLawhorn, Bonnie C Carney, Lauren T Moffatt, Jeffrey W Shupp, Shawn Tejiram

**Affiliations:** The Burn Center at MedStar Washington Hospital Center, Washington, DC; The Burn Center at MedStar Washington Hospital Center, Washington, DC; Georgetown University School of Medicine, Washington, DC; MedStar Health, Washington, DC; The Burn Center at MedStar Washington Hospital Center, Washington, DC; The Burn Center at MedStar Washington Hospital Center, Washington, DC; MedStar Washington Hospital Center, Washington, DC; MedStar Washington Hospital Center, Washington, DC

## Abstract

**Introduction:**

Hyperglycemia is a frequent metabolic disturbance following burn injury, contributing to immune dysregulation, impaired wound healing, and increased infection risk. While glycemic control is routinely monitored in diabetic patients, the relationship between early stress hyperglycemia in non-diabetic burn (NDM) patients, infection, and mortality remains underexplored. This study investigated the association between early hyperglycemia within 48 hours of admission and both infection and mortality in NDM patients.

**Methods:**

Data extracted from the Burn Clinical Quality Program (BCQP) registry (2016-2018) were analyzed. Patients with diabetes were excluded. Blood glucose levels were recorded during the first 24- and 48-hours following admission. Hyperglycemia per BCQP was defined as >250 mg/dL. NDM patients were stratified based on the presence or absence of early hyperglycemia within 48 hours of admission. Primary outcomes included acute respiratory distress syndrome (ARDS), ventilator-associated pneumonia (VAP), central line associate bloodstream infection (CLABSI), and mortality. Multivariable logistic regression assessed associations between early hyperglycemia, infectious complications, and mortality.

**Results:**

Of 2941 NDM burn patients (2088 male (71.0%),White (64.6%), median age of 46 years) that met inclusion criteria, 6.0% (n = 175) developed early hyperglycemia within 48 hours of admission and was associated with younger age (39 v 46 years, *p*=.003) and larger TBSA burns (8.0 v 4.0%, *p*<.0001). NDM patients with early hyperglycemia had significantly higher rates of ARDS (6.3% v 1.2%), VAP (17.2% v 1.8%), and CLABSI (5.7% v 1.1%) (all *p*<.0001). No differences were found in hospital length of stay (LOS, 5 v 5 days, *p*=.78), ICU LOS (6 vs 5 days; *p*=.41), and ventilator days (5.5 vs 3, *p*=.55). Multivariable analysis demonstrated early hyperglycemia independently increased odds of ARDS (OR: 5.56 [95% CI: 2.71-11.42]), VAP (12.04 [7.32-19.80]), and CLABSI (5.98 [2.83-12.67]). Risk of ARDS was highest odds when early hyperglycemia occurred at both 24 hours and 48 hours after admission (9.58 [3.10-29.59]) and 48 hours alone (6.06 [1.74-21.06]). VAP risk peaked with early hyperglycemia in the first 24 hours (16.63 [9.44-29.31]), while CLABSI was associated only with early hyperglycemia (9.06 [3.96-20.72]). Odd of mortality was not associated with early hyperglycemia (0.61 [0.25-1.47]; *p*=.27).

**Conclusions:**

Early hyperglycemia in NDM burn patients is strongly associated with increased risk of ARDS, VAP, and CLABSI, but not mortality. These findings underscore the importance of early glycemic monitoring for timely intervention to mitigate infectious complications.

**Applicability of Research to Practice:**

These findings highlight the need for targeted glycemic management protocols in NDM burn patients and heightened monitoring for infection risk.

**Funding for the Study:**

N/A.

